# Global bibliometric analysis of Tourette syndrome research (1960–2024): trends, collaborations and emerging themes

**DOI:** 10.3389/fneur.2025.1564511

**Published:** 2025-06-06

**Authors:** Zhu Siying, Tian Dong, Tai Xiantao, Xiong Guangyi

**Affiliations:** ^1^Second Clinical Medical College, Yunnan University of Chinese Medicine, Kunming, Yunnan, China; ^2^School of Basic Medical Sciences, Yunnan University of Chinese Medicine, Kunming, Yunnan, China

**Keywords:** Tourette syndrome, bibliometric analysis, global research trends, visualization, collaborative networks, genetic-environmental interaction

## Abstract

**Introduction:**

Tourette Syndrome (TS), a complex neurodevelopmental disorder, has seen a substantial increase in research activity, yet a systematic bibliometric analysis elucidating the global research landscape remains lacking. This study therefore employs bibliometric methods to comprehensively examine the evolution of TS research trends, international collaboration patterns, core contributors, and research hotspots, thereby providing a scientific foundation for future research directions and policy development.

**Methods:**

Based on the Web of Science Core Collection, a topic-based search strategy yielded 4,011 records (1960–2024). Bibliometric analyses were performed using R software and VOSviewer, incorporating annual publication trends, geographical distribution, journal impact metrics (impact factor and H-index), core author collaboration networks, and keyword co-occurrence mapping to assess the structure and dynamics of the research ecosystem.

**Results:**

The bibliometric analysis encompassed 4,011 publications involving 12,860 authors and 5,524 keywords. TS research exhibited a phased growth pattern. Psychiatry, psychology, and neurosciences & neurology emerged as the dominant research domains. While the United States remained the primary contributor, European countries—particularly the United Kingdom, Germany, and Denmark—demonstrated superior international collaboration. *Movement Disorders* proved the most productive journal, whereas *JAMA Psychiatry* held the greatest impact. Leading contributors such as Dr. James F. Leckman and institutions including Yale University showed exceptional research productivity. Over time, research themes have shifted from early emphases on genetics and neuroimaging to recent focuses on patient quality of life and precision interventions, reflecting a trend toward interdisciplinary integration and clinical translation.

**Conclusion:**

Tourette syndrome (TS) research has evolved from descriptive analyses to multidisciplinary integration, yet requires enhanced cross-regional collaboration and application of emerging technologies. Future efforts should prioritize elucidating gene–environment interaction mechanisms, advancing AI-assisted diagnostics, and refining personalized treatment strategies. Concurrently, bridging regional research disparities through global alliances and standardized data platforms is imperative to ensure that scientific discoveries are translated into clinical and societal benefits. Study limitations regarding potential language and database biases underscore the importance of inclusive methodologies in subsequent investigations.

## 1 Introduction

Tourette Syndrome (TS) is a neurodevelopmental disorder characterized by persistent motor and vocal tics for at least 1 year, often accompanied by comorbid neuropsychiatric conditions such as Attention Deficit/Hyperactivity Disorder (ADHD) and Obsessive-Compulsive Disorder (OCD), which complicate diagnosis and management (1–4). The disorder typically manifests between the ages of 2 and 21, with symptom severity peaking in early adolescence. The epidemiology of TS is more complex than previously believed; it was once considered a rare disorder, and some even labeled it as “psychogenic” (5). The prevalence of TS varies depending on its definition, diagnostic criteria, and the methods used in epidemiological studies. While many individuals experience a reduction in tic severity during late adolescence, a significant proportion continue to face persistent tics and associated psychosocial challenges into adulthood (6, 7). TS has a profound impact on academic performance, employment prospects, and quality of life, leading to substantial social and economic burdens, which underscores the need for continued research in this field (8, 9).

Bibliometrics, first introduced by Pritchard in 1969 (10), has become a crucial tool for evaluating interdisciplinary research trends and academic impact. This quantitative approach analyzes publications, citations, and collaboration networks to reveal the structure and dynamics of research fields. Bibliometric tools such as VOSviewer (11) and R-bibliometrix (12) enable the visualization of key themes, influential authors, and emerging trends, offering valuable insights into the evolution of scientific knowledge. In the fields of neuroscience and psychiatry, bibliometric analyses have successfully elucidated the research landscapes of disorders such as autism spectrum disorder (ASD) (13) and Attention Deficit/Hyperactivity Disorder (ADHD) (14), guiding future research directions.

Although research on TS has grown significantly, comprehensive bibliometric analyses that provide an integrated overview of the global research landscape remain limited (15). Most existing studies focus on specific areas, such as pharmacological interventions or genetic research, without offering a synthesis of broader research trends and knowledge gaps (16, 17). Given the importance of bibliometric analyses in mapping research landscapes and identifying emerging themes (18, 19), there is a clear need for a comprehensive bibliometric study on TS to guide future research directions and foster collaboration. To address this gap, this study conducts a bibliometric analysis of TS-related publications spanning 1960 through 2024, with particular emphasis on publication trends, leading contributors, and emerging research hotspots. By offering a detailed overview of the current state of TS research, this paper aims to highlight opportunities for innovation and guide future scientific exploration.

## 2 Methods

### 2.1 Literature retrieval strategy

Selecting an appropriate data source is critical for bibliometric analysis. The Web of Science (WOS) database is widely recognized as a premier choice for bibliometric studies due to its extensive coverage, high-quality indexing, and robust citation analysis capabilities (20). This study employs bibliometric methods to map the global TS literature. On April 30, 2025, we retrieved 7,699 records from the Web of Science Core Collection using a targeted query—TS = (“Tourette* Syndrome” OR “Gilles de la Tourette” OR “motor tic*” OR “vocal tic*” OR “coprolalia”)—to focus on core TS terminology and avoid dilution by broader “tic disorder” entries. We then excluded non-TS tic disorders (“Tic Disorder,” “Chronic Tic”), applied a filter to remove misleading terms (“tick,” “Lyme”), and restricted results to English-language articles and review articles to ensure consistency and peer-reviewed quality. Next, we discarded 35 records from the incomplete year 2025 and two isolated entries from 1943 and 1953 (Data verification revealed that the literature records from 1943 and 1953 exhibited a 100% missing rate for the following fields: Abstract, Corresponding Author, Author Keywords, and Keywords Plus). By including publications from 1960 to 2024, the bibliometric analysis offers a comprehensive, continuous, and systematic view of the long-term trends and evolutionary phases in TS research.

**Figure 1:**
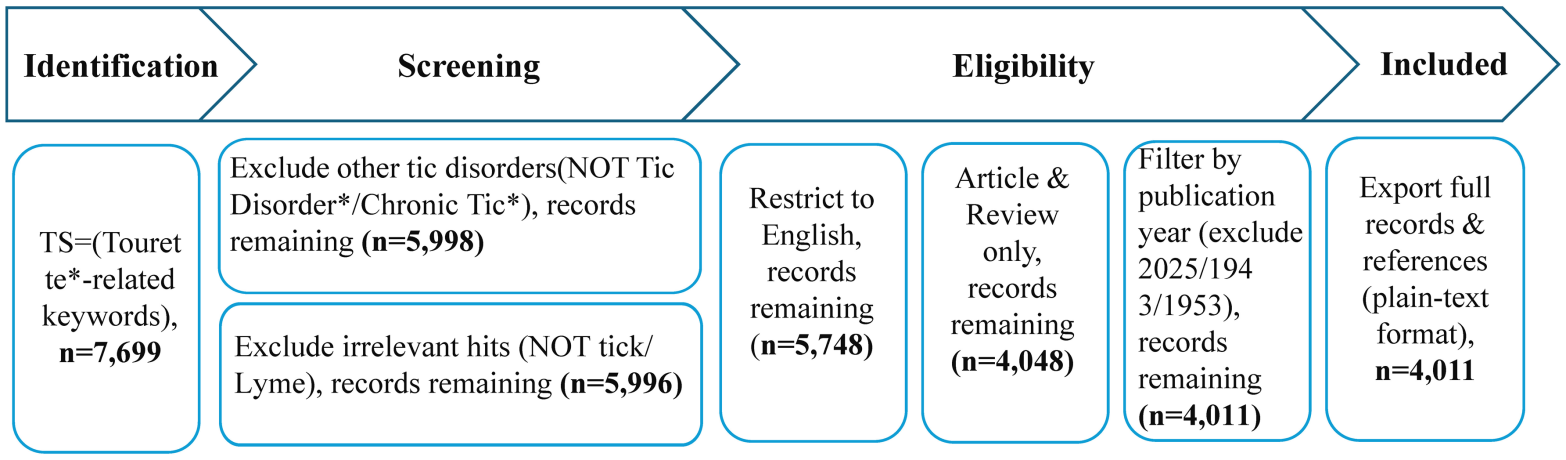
Literature search and screening flowchart.

Through multiple iterations of search strategy optimization, a balance between recall and precision was ensured. All data cleaning steps (such as criteria for record exclusion) were transparently presented in a flowchart (Figure 1), enhancing methodological reproducibility. The refined dataset comprised 4,011 records, all exported in “Plain Text” format with “Full Records and Cited References” for downstream bibliometric analysis.

### 2.2 Bibliometric analysis

Bibliometric analysis typically follows a structured five-step approach, including study design, data collection, analysis, visualization, and interpretation (12). Table 1 illustrates this methodological framework, which ensures a systematic and comprehensive analysis of research data.

**Table 1:** Phases and components of the bibliometric analysis methodology.

Phase	Component	
Phase1 Study Design	Topic Selection	
	Research Question	
	Database Selection	
Phase2 Data Collection	Data Loading	
	Data Converting	
Phase3 Analysis	Document Attribute Matrix Creation	Data Reduction
		Network Matrix Creation
Phase4 Visualization	Mapping	
Phase5 Interpretation	Network Map	
	Historiograph	

The bibliometric analysis methodology comprised five phases, each consisting of specific components.

In the study design phase, we selected TS as the research topic and chose the Web of Science (WOS) Core Collection as the data source. During the data collection phase, we performed a comprehensive and precise search using a specific search formula in WOS, ensuring scientific rigor and reliability by selecting peer-reviewed articles and reviews, which yielded 4,011 documents.

No duplicates were identified due to the high-quality indexing of the Web of Science Core Collection, which ensures unique records. All records were imported using Biblioshiny Web and converted into Bibliometrix R data and Excel formats for further analysis. Missing values in the exported Excel spreadsheet (including cited references, author affiliations and countries, DOIs, and journal impact factors) were filled, and information on countries, institutions, and journals was extracted to assess their impact. Additionally, key bibliometric indicators such as publication year, publication count, author count, and keyword count were extracted using Biblioshiny, which facilitated the generation of descriptive statistics. Moreover, data on national publication output, collaboration patterns, and high-frequency keyword trends were exported for subsequent visualization of relevant thematic maps. Tidyverse (ggplot2) was employed to create high-quality graphics and visualizations for the temporal evolution and key themes in TS research. Additionally, VOSviewer was used to construct co-citation networks, keyword co-occurrence networks, and collaboration networks, revealing key themes and collaboration patterns within the research field.

We applied Bradford’s law to classify journals in the TS field into “core” and “dispersed” zones (21). Journals were ranked in descending order by their number of published articles, and the cumulative article count (4,011 articles across 991 journals) was calculated. The total corpus was then partitioned into three equal segments of approximately 1,337 articles each. The smallest set of journals whose combined output reached the first segment was defined as the core zone (Zone 1); the next set of journals accounting for the second segment comprised Zone 2; and the remaining journals constituted the peripheral zone (Zone 3). In our analysis, the core zone comprised 36 journals, which is consistent with the minimal core size predicted by Bradford’s distribution. This threshold-based approach allowed us to identify a small set of highly productive core journals and to quantify the contributions of the larger body of less-productive journals.

The results of our data analysis and visualization, including insights from Bradford’s law, will be detailed in the subsequent sections. This study employed a robust methodology to ensure the validity of bibliometric insights and enhance our understanding of global trends in TS research.

## 3 Results

The preliminary findings of the bibliometric analysis offer a detailed overview of the current landscape in TS research. After applying document type filters and excluding irrelevant records, a total of 4,011 papers published between 1960 and 2024 were included in the study. The analysis examined publication trends, geographical distribution, journal contributions, author influence, and keyword patterns, providing insights into the developmental trajectory, global distribution, and key research areas within TS. Detailed discussions of these aspects, including the methods and tools used for analysis, are provided in the following sections.

### 3.1 Descriptive bibliometric analysis

The descriptive analysis provides a comprehensive overview of the research landscape on TS (Table 2), encompassing 4,011 documents published between 1960 and 2024, reflecting a mature yet evolving field. These studies are disseminated across 911 diverse sources, indicating a broad and interdisciplinary approach. The analysis identifies 5,524 author keywords and 6,482 Keywords Plus from references, highlighting the wide range of subtopics and methodologies explored in TS research. The collaborative nature of the field is evident, with an average of 3.21 authors and 5.43 co-authors per document, suggesting a robust and interconnected research community. Furthermore, the average citation rate of 46.58 citations per document underscores the significant influence and recognition of TS research within the academic community.

**Table 2:** Descriptive analysis of TS-research data.

Main information	Description	Value
Documents	Total number of documents	4,011
Sources	The frequency distribution of sources as journals, books, etc.	911
Timespan	Years of publication	1960–2024
Auther’s keywords (DE)	Total number of author’s keywords	5,524
Keywords plus (ID)	Total number of phrases that frequently appear in the title of an article’s references	6,482
Authors	Total number of authors	12,860
Authors appearances	The authors’ frequency distribution	21,768
Authors of single-authored documents	The number of single authors per articles	332
Authors per document	Average number of authors in each document	3.21
Co-Authors per documents	Average number of co-authors in each document	5.43
Average citations per documents	Average number of citations in each document	46.58

### 3.2 Publication trends

**Figure 2:**
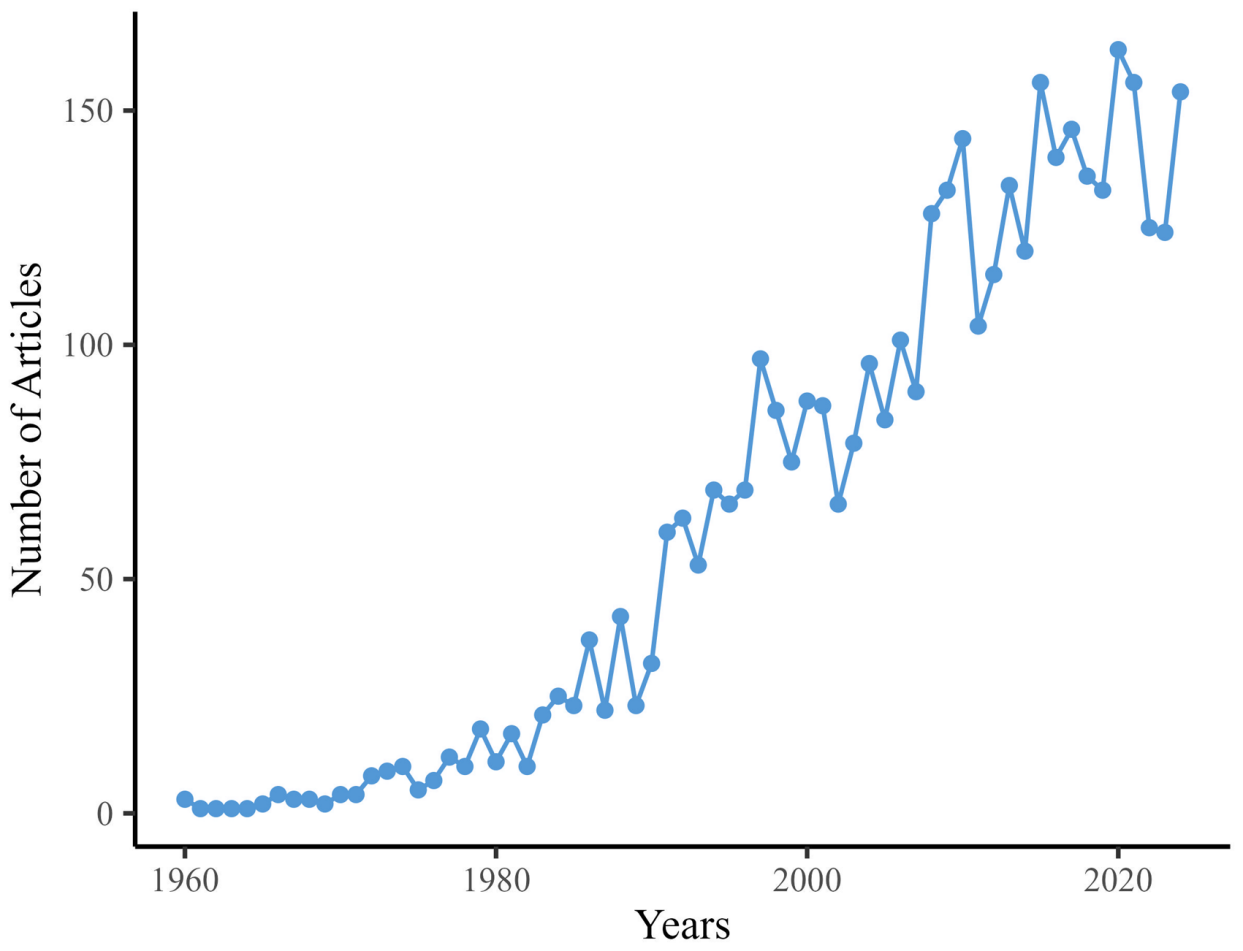
Annual publication trends in TS-research (1960–2024).

Figure 2 delineates the annual publication trends in TS research. Output remained minimal (≤4 articles/year) throughout the 1960s, first reaching double digits in 1974 (10 articles). Steady growth characterized the 1980s, peaking at 42 articles in 1988. A pronounced acceleration occurred during the 1990s, culminating in 97 articles by 1997. The early 2000s saw fluctuations between 66 and 101 annual publications, followed by sustained expansion from 2008 onward, with output exceeding 120 articles/year after 2013 and reaching its zenith in 2020 (163 articles). A transient decline to 124 articles in 2023 preceded a rebound to 154 in 2024. This pattern of escalating output, punctuated by periodic milestones, aligns with the developmental trajectory of an evolving research domain.

### 3.3 Research areas

**Figure 3:**
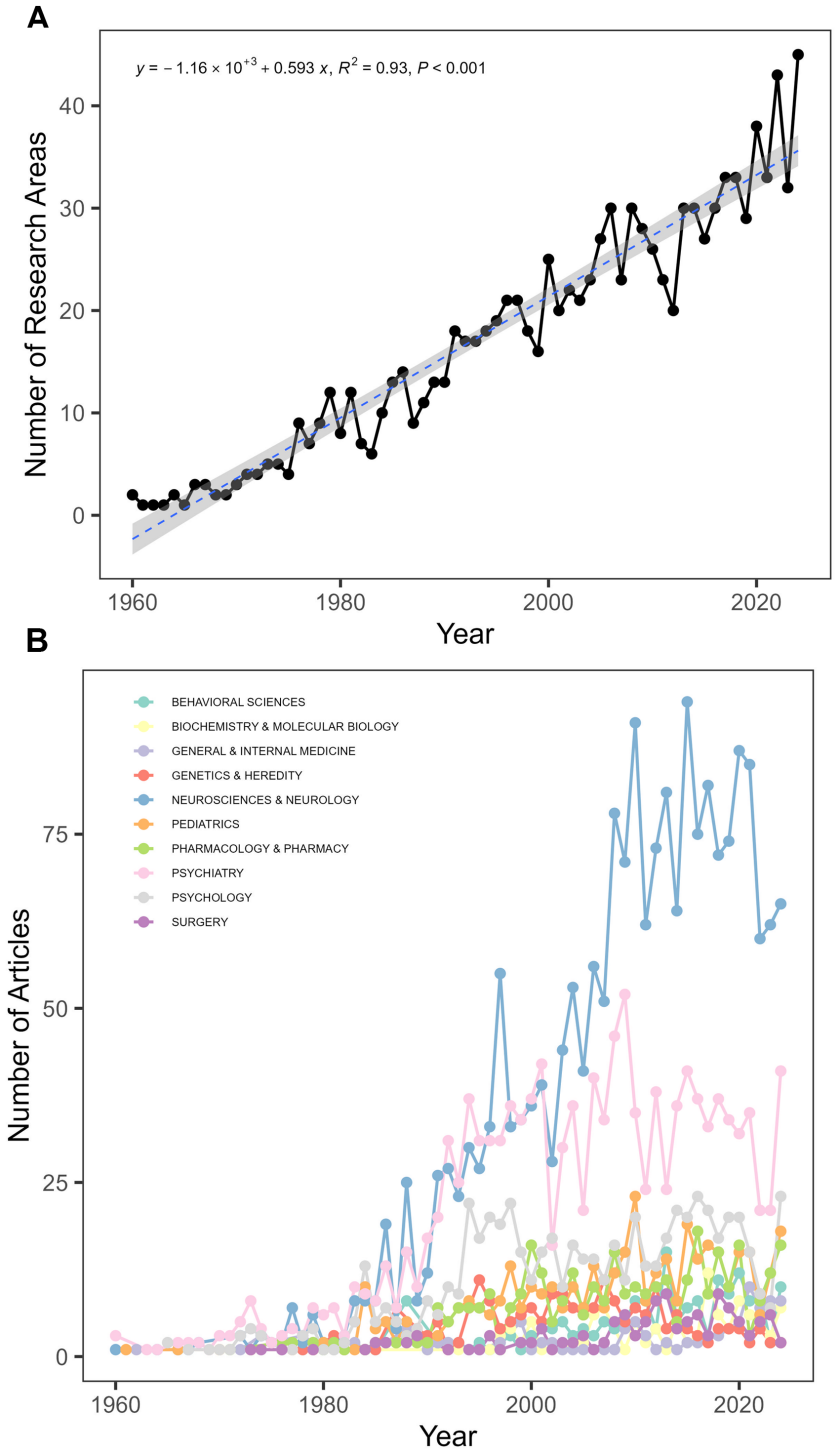
Temporal evolution and productivity of TS research areas. **(A)** Temporal evolution of research areas; **(B)** Top 10 most productive research areas.

Following the classification framework of Clarivate Analytics, each paper in the Web of Science (WOS) database is allocated to one or more research areas. Figure 3A illustrates the temporal evolution of TS research areas, showing an expansion from 2 fields in 1960 to 45 fields in 2024. Figure 3B presents the top 10 most productive research domains in TS research, including behavioral sciences, biochemistry and molecular biology, general and internal medicine, genetics and heredity, neurosciences and neurology, pediatrics, pharmacology and pharmacy, psychiatry, psychology, and surgery. These fields constitute 91.25% of the total TS-related publications (3,660 out of 4,011). Over the period from 1960 to 2024, psychiatry, psychology, and neurosciences and neurology consistently emerged as the leading research areas, with neurosciences and neurology achieving its highest publication volume in 2015 (n = 91).

### 3.4 Research countries

**Figure 4:**
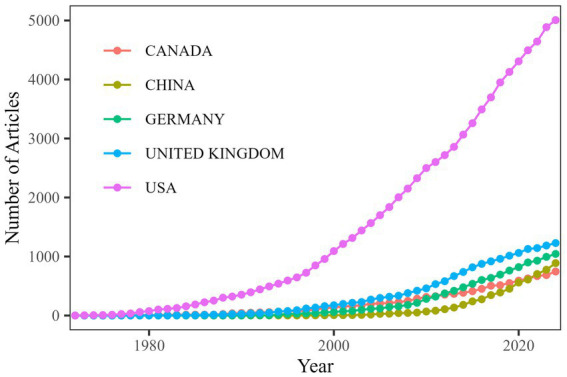
Annual publication trends by country. Publication counts are based on author nationality; multi-country collaborations are counted for each contributing nation.

**Figure 5:**
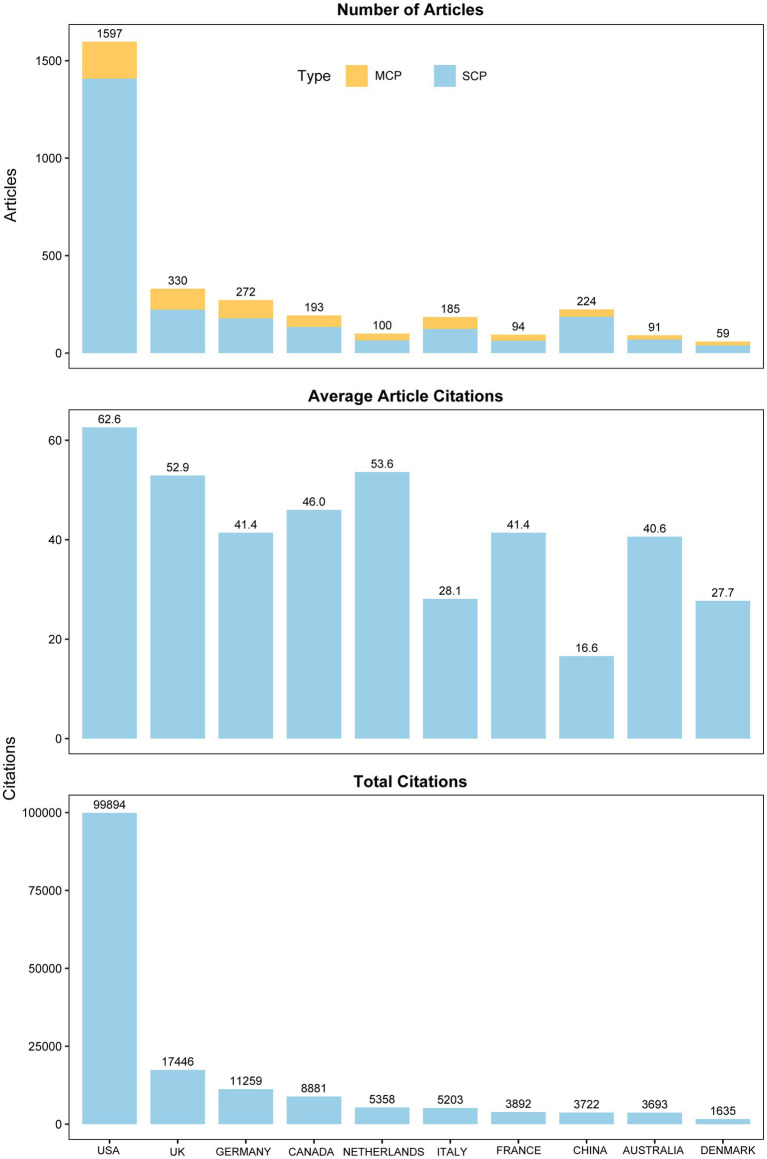
Research performance of top 10 countries: collaboration types and citations. SCP, Single Country Publication; MCP, Multinational Cooperative Publication.

**Figure 6:**
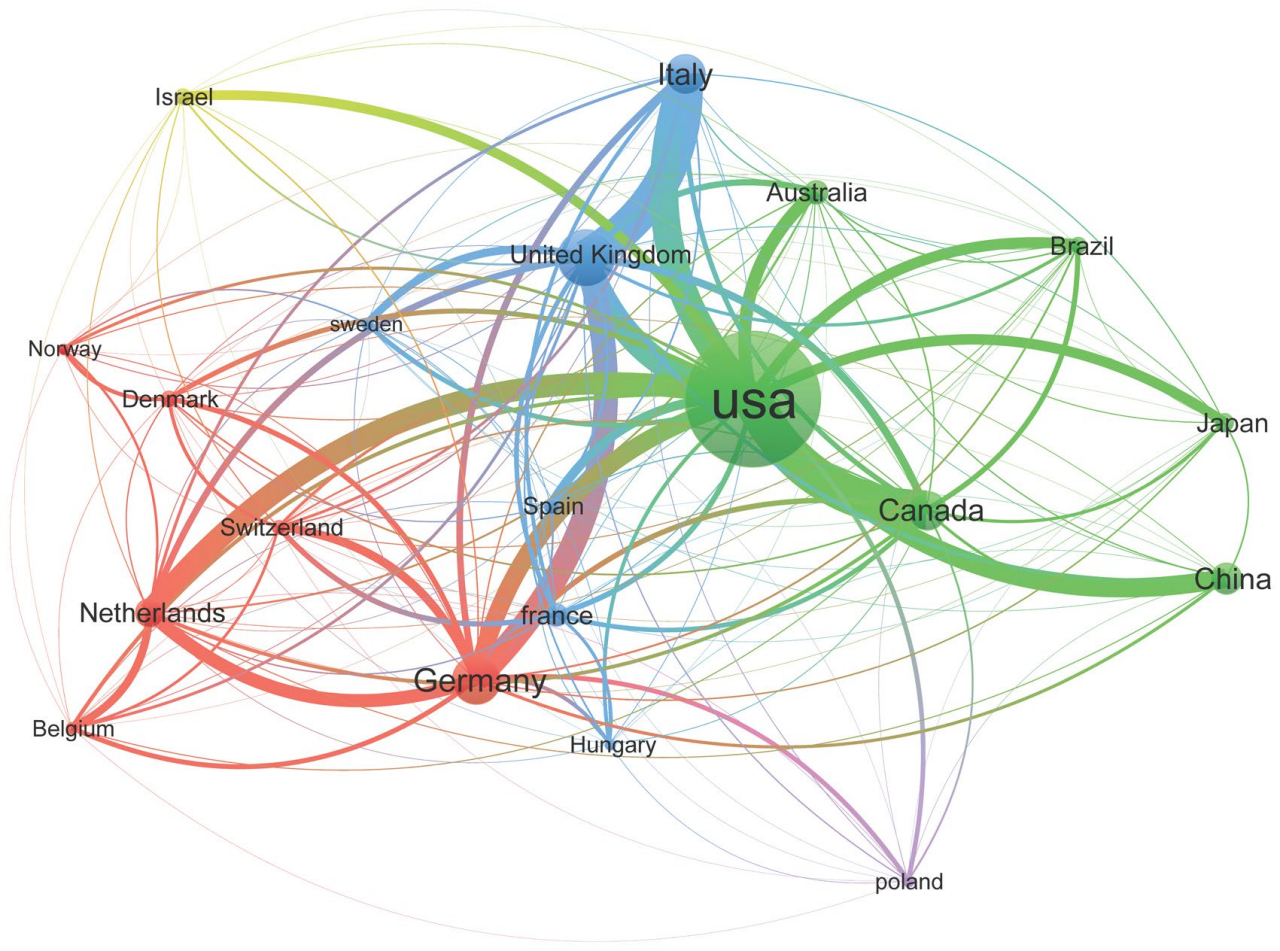
Global collaboration network.

The research findings indicate that since 1960, a total of 61 countries have participated in studies on TS, with the United States consistently leading in publication numbers and maintaining its dominant position (Figure 4). The top five countries in terms of scientific output are the United States (*n* = 1,597), the United Kingdom (*n* = 330), Germany (*n* = 272), China (*n* = 224), and Canada (*n* = 193). After analyzing the data, we assessed the research performance of the top 10 countries, including their research cooperation models, total citations, and average citations per article (Figure 5). The first part presents the research cooperation types of each country, including SCP (Single Country Publication) and MCP (Multinational Cooperative Publication). The United States, with the highest number of publications (*n* = 1,597), primarily consists of single-country collaborations (*n* = 1,408, 88.2%). In contrast, European nations exhibit higher proportions of multinational collaborations (MCP), with the United Kingdom (32.4%), Germany (34.6%), Italy (33%), France (33%), and particularly the Netherlands (35%) demonstrating the most prominent engagement patterns. These elevated MCP rates collectively signify stronger intra-regional academic collaboration trends within Europe. The average citation count per article reflects the quality and influence of each country’s research outcomes. United States has the highest average citation count (*n* = 62.6), followed by the Netherlands (*n* = 53.6) and the United Kingdom (*n* = 52.9). The United States leads in total citations with 99,894, demonstrating its absolute advantage in research. The United Kingdom and Germany rank second and third with 17,446 and 11,259 citations, respectively. Notably, despite China’s high publication output of 224 articles, its total citation count is relatively low at 3,722, with an average of 16.6 citations per article, highlighting a gap in international influence compared to research powerhouses in Europe and America. In terms of global collaboration (Figure 6), the United States has the highest number of international collaborations with 54 connections. The United Kingdom follows closely with 48 connections, while Germany ranks third at 43. Canada and the Netherlands each demonstrate 39 collaborations, and Italy completes the network with 37 connections.

### 3.5 Leading source journals

**Figure 7:**
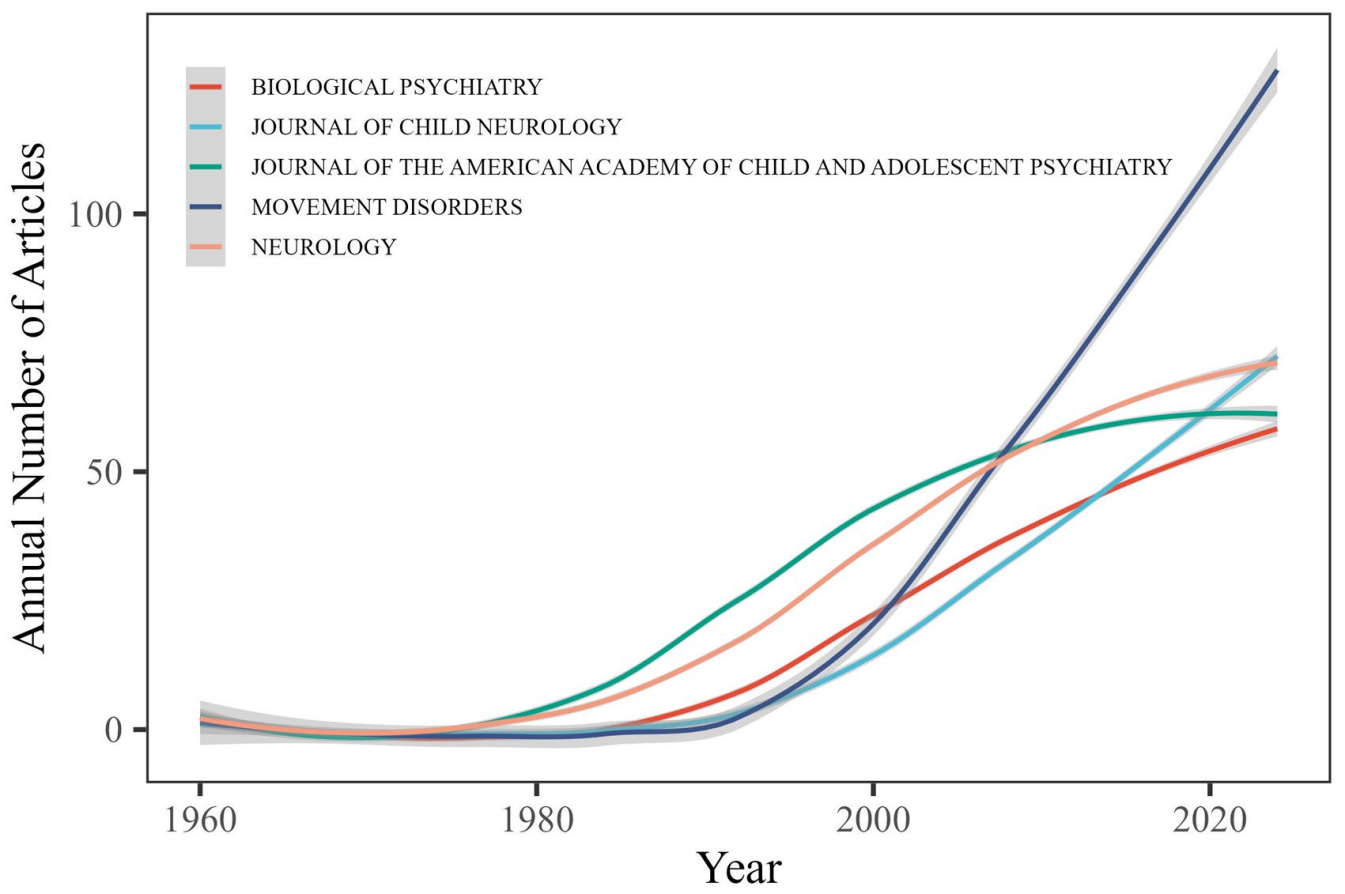
Annual publication trends in Top 5 journals.

TS research findings are disseminated across 991 journals, yet their publication patterns closely follow Bradford’s Law of Scattering, displaying a marked core–periphery structure. Specifically, the top five outlets—*Movement Disorders* (114 papers), *Neurology* (22), *Journal of Child Neurology* (23), *Journal of the American Academy of Child and Adolescent Psychiatry* (24) and *Biological Psychiatry* (25)—have published a total of 369 papers (Figure 7). In contrast, 540 journals (54.4%) each published only one TS article, and 894 journals (90.2%) published 10 or fewer. This long-tail distribution is characteristic of a mature yet dispersed research field.

Regarding local citation impact, JAMA Psychiatry demonstrates the highest citation frequency among TS publications (Table 3). The top 10 journals identified through local citation analysis include multiple journals recognized as core sources for TS research, such as Neurology, Movement Disorders and Biological Psychiatry. These journals have significantly contributed to advancing TS research through their influential scholarly output.

**Table 3:** Top 10 journals based on influence metrics.

Source	TC	NP	IF	h_index	g_index	PY_start
JAMA Psychiatry*	8,376	40	22.5	36	40	1966
Neurology*	7,614	70	8.4	46	70	1966
Movement Disorders*	5,491	114	7.4	43	69	1990
Biological Psychiatry*	5,326	57	9.6	36	57	1987
JAACAP*	5,222	62	9.2	42	62	1973
Journal of Child Psychology and Psychiatry*	4,976	26	6.5	20	26	1992
Brain*	4,927	36	11.9	33	36	1991
American Journal of Psychiatry*	4,683	30	15.1	27	30	1960
Molecular Psychiatry*	2,873	32	9.6	24	32	1996
JNNP*	2,812	44	8.8	30	44	1973

JAACAP, Journal of the American Academy of Child and Adolescent Psychiatry; JNNP, Journal of Neurology, Neurosurgery and Psychiatry; X*, the core journal of TS research as defined by Bradford Law; TC, total citations; NP, number of papers; PY-start, Year of first publication of articles. IF, impact factor in 2023.

### 3.6 Most influential authors and organizations

The H-index serves as a prominent metric to gage a researcher’s academic impact, taking into account the citation frequencies of their published works (26). In this study, we determined the H-index for every author involved in the 4,011 TS research articles, irrespective of their ranking in the author sequence. The authors with the highest H-indices include Leckman J. F. with 107 publications, Robertson M. M. with 98, Singer H. S. with 76, as detailed in Table 4. Leckman J. F., boasting an M-index of 1.349, stands out as the leading author in terms of both publication volume and citation metrics. Dr. James F. Leckman, a distinguished professor at Yale University School of Medicine, is renowned for his pioneering work in the neurobiology of TS and related developmental disorders. His research has significantly advanced our understanding of the genetic and environmental factors influencing TS, and he has been instrumental in developing comprehensive assessment tools for tic disorders (27–29). Among the top 10 influential researchers, eight are affiliated with institutions in the United States, one in the United Kingdom, and one in Canada. Overall, our analysis encompassed 4,011 papers, which involved a total of 12,860 authors, among whom 332 produced single-authored works. With an average of 5.43 co-authors per paper, this suggests a strong inclination toward collaborative efforts in TS research.

**Table 4:** Top 10 authors based on influence metrics.

Author	TC	NP	h_index	g_index	m_index	PY_start	Country
Leckman J. F.	12,187	107	58	107	1.349	1983	USA
Robertson M. M.	6,979	98	47	82	1.175	1986	UK
Singer H. S.	6,588	76	43	76	0.896	1978	USA
Pauls D. L.	5,268	60	39	60	0.867	1981	USA
Jankovic J.	3,749	67	37	61	0.86	1983	USA
Cohen D. J.	5,070	51	36	51	0.766	1979	USA
Comings D. E.	4,782	50	35	50	0.778	1981	USA
Cavanna A. E.	2,713	83	31	49	1.632	2007	UK
Biederman J.	5,357	35	29	35	0.725	1986	USA
Peterson B. S.	4,009	36	29	36	0.853	1992	USA

Country: Country where the authors’ primary contributions were made.

Table 5 presents the top 10 institutions ranked by academic productivity and citation impact. Yale University leads with 218 papers and 18,521 citations, reflecting both high productivity and broad influence. Harvard University, despite fewer publications (116 papers), demonstrates exceptional scholarly impact through 14,291 citations. Johns Hopkins University ranks third with 122 papers and 9,391 citations, indicating sustained research engagement. Collectively, these institutions exemplify global leadership in research and academic excellence, with the United States prominently featuring as a hub for scientific inquiry.

**Table 5:** Top 10 influential institutions in TS research.

Organization	Papers	Citations	Country
Yale University	218	18,521	United States
Harvard University	116	14,291	United States
Johns Hopkins University	122	9,391	United States
Massachusetts General Hospital	68	8,274	United States
University College London	169	7,930	United Kingdom
UCLA	62	5,747	United States
University of Toronto	87	5,314	Canada
NIMH	57	5,224	United States
University of Utah	31	4,038	United States
Baylor College of Medicine	85	3,700	United States

UCLA, University of California, Los Angeles; NIMH, National Institute of Mental Health.

### 3.7 Most influential papers

This study incorporated 121,908 references, with citation counts defined by local citations (LC) (30)—that is, the number of times each work was cited within our dataset—and Table 6 presents the 10 most influential papers ranked by LC.

**Table 6:** Top 10 papers according to the local citation score.

Paper	DOI/ PMID	LC	NLC	Year
PAULS DL, 1986, ARCH GEN PSYCHIAT	10.1001/archpsyc.1986.01800120066013	285	9.51	1986
FREEMAN RD, 2000, DEV MED CHILD NEUROL	10.1017/s0012162200000839	221	24.84	2000
COMINGS DE, 1987, AM J HUM GENET	2,890,294	218	3.14	1987
LECKMAN JF, 1998, PEDIATRICS	10.1542/peds.102.1.14	181	15.69	1998
PETERSON BS, 1998, ARCH GEN PSYCHIAT	10.1001/archpsyc.55.4.326	178	15.43	1998
PETERSON BS, 2003, ARCH GEN PSYCHIAT	10.1001/archpsyc.60.4.415	169	12.55	2003
ALBIN RL, 2006, TRENDS NEUROSCI	10.1016/j.tins.2006.01.001	166	15.48	2006
BOHLHALTER S, 2006, BRAIN	10.1093/brain/awl050	164	15.29	2006
KALANITHI PSA, 2005, P NATL ACAD SCI USA	10.1073/pnas.0502624102	159	10.61	2005
SINGER HS, 1993, NEUROLOGY	10.1212/wnl.43.5.950	146	7.91	1993

LC, Local Citations; Citation count within the TS-focused literature dataset; NLC, Normalized Local Citations; LC divided by the annual average LC of TS papers, controlling for citation accumulation over time.

The most cited paper, by Pauls et al. (31), employed semi-structured interviews of TS probands and their first-degree relatives to demonstrate that obsessive-compulsive disorder (OCD) aggregates with TS in families, providing the first clear evidence of shared heritable susceptibility and laying the methodological groundwork for subsequent genome-wide linkage and SLITRK1 candidate-gene studies (32) as well as revisions to DSM comorbidity criteria; Freeman et al. (33) analyzed a multicenter cohort of 3,500 TS patients from 22 countries to characterize sex ratio, age at onset, comorbidity (88% ADHD), and regional variation, supplying robust epidemiological data that have informed international practice guidelines and the design of multicenter intervention trials; Comings et al. (34) compared 246 TS patients with 47 controls using DSM-III–based questionnaires to quantify familial co-occurrence of ADHD, learning disorders, and school difficulties, establishing a phenotypic screening paradigm subsequently adopted in candidate-gene investigations; Leckman et al. (27) conducted longitudinal follow-up of TS children, identifying a peak in tic severity during adolescence followed by marked remission in early adulthood, thereby refining prognostic counseling and optimizing intervention timing; Peterson et al. (35) used fMRI in 22 adult TS patients to compare neural activity during voluntary tic suppression versus free expression, revealing enhanced activation in basal ganglia–thalamocortical regions inversely related to symptom severity and guiding TMS/DBS target selection (36, 37); in a subsequent structural MRI study (38), quantitative analyses demonstrated reduced caudate nucleus volumes in TS subjects relative to controls, with volumetric reductions correlating with comorbid OCD and symptom persistence, thereby validating a neuroanatomical biomarker and motivating machine-learning–based prognostic models; Albin & Mink (39) synthesized evidence for dysregulation of the cortico–striato–thalamo–cortical circuitry and interactions among dopamine, GABA, and glutamate systems to propose a unified pathophysiological model that has underpinned VMAT2 inhibitor development and neuromodulation approaches (40, 41); Bohlhalter et al. (42) demonstrated via task-based fMRI that TS patients exhibit weakened fronto–parietal–basal ganglia connectivity during motor inhibition tasks, highlighting network-level deficits that have informed network-guided TMS protocols and subtype stratification; Kalanithi et al. (43) provided the first postmortem evidence of reduced parvalbumin-positive GABAergic interneurons in the basal ganglia of TS cases, corroborating an inhibitory signaling deficit hypothesis and directly supporting GABA-targeted therapeutic development (44, 45); and Singer (46) assessed adult TS patients’ neuropsychological and motor functions, showing that cognitive and attentional deficits often persist despite tic remission, thereby emphasizing the need for lifelong neuropsychological monitoring and individualized rehabilitation plans.

Together, these studies span family-based epidemiology, natural history, functional and structural neuroimaging, and pathophysiological synthesis, mapping a comprehensive research trajectory from genetic susceptibility to clinical intervention in TS.

### 3.8 Analysis of prominent research trends

**Figure 8:**
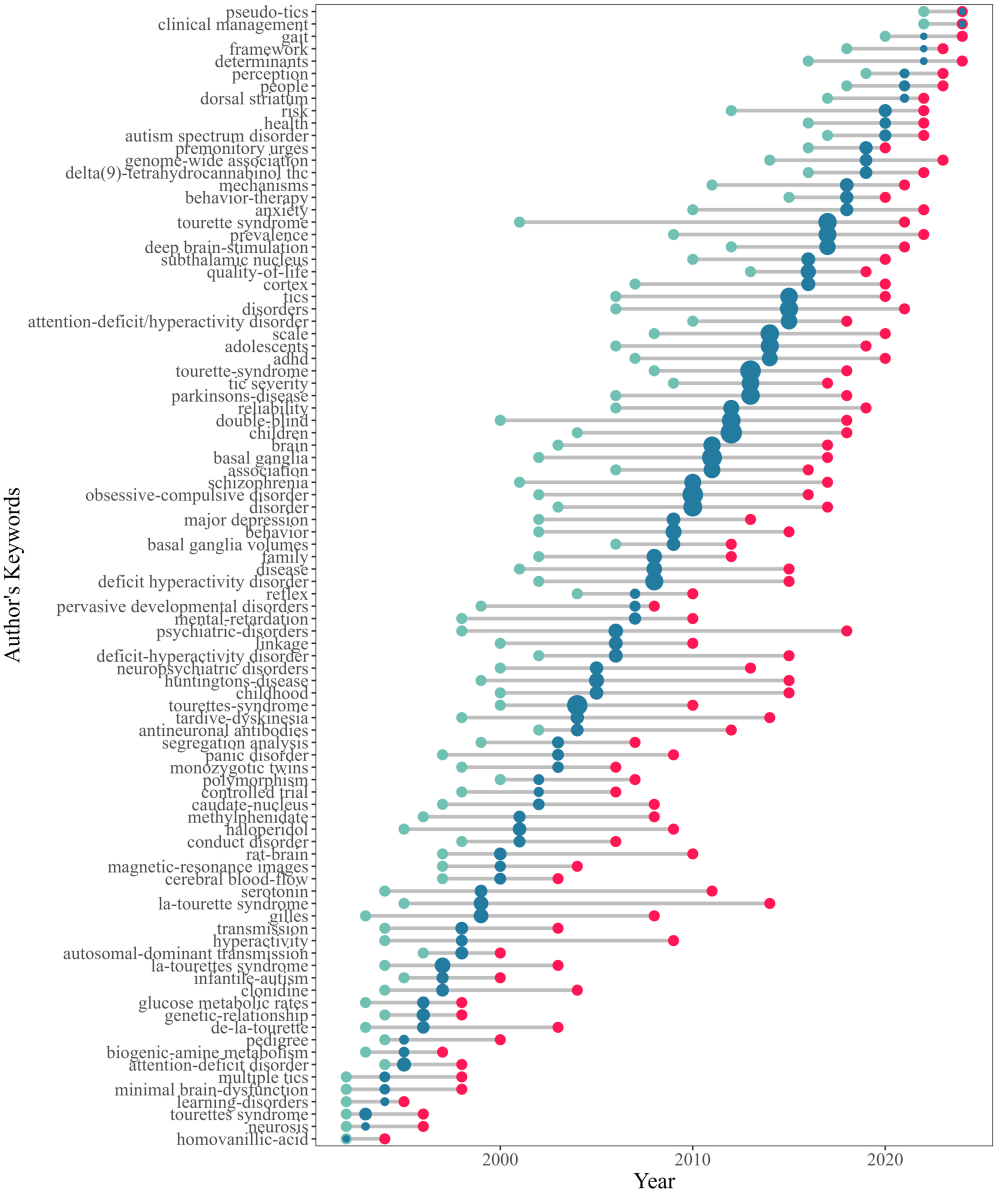
Temporal trends in TS research keywords.

This study employed bibliometric methods to analyze research trends in TS. Figure 8 illustrates the temporal trends in author keywords, with the x-axis representing publication years and the y-axis showing keywords. Each keyword’s publication years are represented by three quartiles: green dots indicate the first quartile, blue dots the median publication year, and red dots the third quartile. The size of the blue dots corresponds to keyword frequency, with larger dots indicating higher frequencies.

The analysis of keywords reveals the top 10 most frequently occurring terms in TS research: Tourette syndrome, Children, obsessive-compulsive disorder, basal ganglia, disorder, double-blind, scale, Parkinsons-disease, disorders and adolescents. Among these, ‘Tourette syndrome’ stands out as a core topic, reflecting its central role in the TS research domain.

The bibliometric analysis delineates five temporal phases in TS research, reflecting evolving methodological and conceptual priorities. Initial investigations (mid-1980s–1991) focused on establishing diagnostic criteria (DSM-III) (47, 48) and epidemiological foundations, with Tourette syndrome, family aggregation (31, 49), and prevalence (50, 51) as key terms. Subsequent clinical phenotyping efforts (1992–1996) prioritized symptom characterization, emphasizing multiple tics and comorbidities like ADHD (46, 52, 53), alongside early neurochemical studies of dopaminergic pathways (25, 54, 55).

The molecular genetics era (1997–2003) saw advancements in candidate gene studies (32, 56, 57) (e.g., SLITRK1 and HDC variants linked to synaptic pruning and histaminergic dysfunction) and structural neuroimaging (35, 38) (magnetic-resonance imaging), alongside immunological hypotheses involving antineuronal antibodies (58, 59). From 2004 to 2014, circuit-level models dominated, driven by deep brain stimulation (60–62) targeting the subthalamic nucleus and dorsal striatum (63, 64), supported by genome-wide association studies (24, 65, 66) integrating genetic risks with cortico-striato-thalamo-cortical circuit abnormalities. Recent research (2015–2024) prioritizes patient-centered outcomes (67), marked by quality-of-life (23, 68, 69) metrics, behavior-therapy for premonitory urges (70), and trials of delta (9)-tetrahydrocannabinol (Δ9-THC) (22, 71). Emerging challenges include differentiating organic tics from pseudo-tics and addressing autism spectrum disorder comorbidity (72, 73). Methodologically, machine learning applications and clinical management frameworks now aim to unify genetic, environmental, and neural circuit insights into actionable care models. This progression illustrates TS research’s shift from descriptive nosology to mechanism-driven, functionally oriented paradigms.

## 4 Discussion

### 4.1 From descriptive epidemiology to precision science

The trajectory of TS research publications reveals a field that has steadily evolved from descriptive epidemiology to sophisticated, patient-centered science. Prior to 1990, annual outputs were modest, reflecting TS’s absence from mainstream nosology; the advent of DSM-III (74) and the first family-aggregation (31, 75) studies in the mid-1980s marked the first sustained uptick as investigators began to quantify heritable risk. The 1990s saw another acceleration, driven by the adoption of MRI and fMRI and landmark longitudinal cohort analyses that illuminated basal-ganglia circuitry and tic-suppression mechanisms. A more pronounced surge in the early 2000s coincided with the rollout of genome-wide linkage and association studies, and the emergence of CSTC circuit models (76), which underpinned the first clinical trials of deep brain stimulation and transcranial magnetic stimulation. Publication rates peaked around the release of DSM-5 (77, 78) in 2013—when expanded diagnostic categories and comorbidity specifiers broadened the scope of inquiry—and then plateaued at a high level as the community embraced standardized quality-of-life measures, digital biomarkers, and new pharmacotherapies such as VMAT2 inhibitors (41). The brief dip in 2020–2021 likely reflects the pandemic’s interruption of in-person research, but the rapid rebound demonstrates the field’s agility in deploying telehealth and remote assessment tools. Together, these inflection points underscore how evolving diagnostic frameworks, methodological innovations, and clinical imperatives have shaped TS research into the multidisciplinary endeavor it is today.

### 4.2 Collaborative capacity, challenges, and future synergies in TS research

The concentration of TS research within a handful of countries and institutions highlights both remarkable capacity and untapped opportunities for collaboration. The United States’ leading position in publication volume and citation impact stems from its abundant funding and well-established clinical–research infrastructure; however, its evaluation system—which places greater weight on high-impact domestic papers than on multinational projects—may inadvertently discourage cross-border partnerships despite ample resources (30, 31). In contrast, European nations such as the United Kingdom, Germany and Denmark benefit from EU initiatives like Horizon Europe, which explicitly reward multinational consortia and have driven their higher proportions of internationally coauthored work and strong citation metrics. This contrast suggests that, while U. S. teams have propelled foundational advances in TS genetics and circuitry, a more concerted engagement with global networks could further enrich study populations, foster methodological innovation and accelerate the translation of mechanistic insights into diverse clinical settings. Facilitating joint grant schemes, shared data platforms and co-mentorship between North American centers and emerging hubs would help distribute expertise more evenly and build a truly global TS research community.

China’s rapid ascent into the top five producers of TS literature reflects successful capacity building, yet its comparatively modest citation rate signals room to enhance study design, target leading journals and forge strategic international alliances. Likewise, burgeoning research pockets in Canada and Australia stand to gain from closer collaborations with established laboratories.

At the individual level, the enduring influence of investigators at Yale, Harvard and Johns Hopkins—particularly figures such as Leckman, Robertson and Singer—has set high benchmarks for cohort size, longitudinal follow-up and translational scope; nevertheless, nurturing a new generation of scholars across diverse geographic and career stages will be vital to sustain this momentum.

Looking forward, deliberate expansion of collaborative frameworks is essential. Multinational genomics consortia, standardized neuroimaging repositories and shared clinical-trial networks can unify efforts, while partnerships between high-output centers and emerging research communities can drive cultural sensitivity and inclusivity in study design (17). By reinforcing these global alliances, the field can ensure that advances in TS research benefit patients everywhere.

### 4.3 Core journal concentration: benefits, drawbacks, and future directions

The concentration of TS research in a small set of core journals—following Bradford’s Law—has clear benefits and drawbacks. On the upside, specialist outlets such as Movement Disorders and Neurology uphold rigorous peer review and ensure that major advances [for example, Yale’s contributions to genetic research and key imaging studies on TS have been pivotal (31, 32, 43)] reach the right audience quickly. On the downside, more than half of all journals publish only one TS paper, which means that useful innovations—like early smartphone apps for automated tic tracking or small telehealth pilot programs—can slip under the radar when they appear outside the core literature.

Looking ahead, balancing depth and breadth will be essential. Leading journals can help by dedicating space to both specialized studies and practical innovations—like testing AI tools that spot tic patterns in home videos, or refining apps that help track symptoms between clinic visits. We also need smarter ways to connect findings from different fields. Envisioning a shared online platform where engineers developing motion sensors collaborate with therapists implementing school-based interventions may facilitate a deeper understanding of why certain treatments demonstrate greater effectiveness in real-world settings. At the same time, annual review collections or simple online consortia could bring together findings from peripheral outlets, whether they describe wearable sensors tested in engineering journals or community-based support models reported in rehabilitation publications. Finally, to close geographic gaps, the TS community should back regional registries (for example, in Latin America or Southeast Asia) and fund translation and validation of key rating scales in multiple languages. These steps will help ensure that TS research remains both rigorous and relevant to patients and clinicians worldwide.

### 4.4 Gene–environment interactions and translational research in Tourette syndrome: from mechanistic insights to precision prevention

The evolving focus of TS research—from mapping cortico-striatal circuits to decoding genetic risks and ultimately improving patients’ daily lives—mirrors both scientific breakthroughs and societal demands for holistic care. Seminal discoveries include SLITRK1—whose rare variants disrupt synaptogenesis and neural circuit connectivity—and HDC, the rate-limiting enzyme in central histamine synthesis, together anchoring TS pathophysiology in both neurodevelopmental and neurochemical pathways (79–81).

Increasing emphasis on quality-of-life outcomes in neurological disorders, including TS, underscores the need to translate laboratory insights into clinical and public health contexts. A central challenge is understanding how genetic predispositions interact with environmental exposures in TS. Epidemiological observations—for instance, rising TS incidence in urban populations and in post-pandemic cohorts—suggest that modifiable environmental factors (e.g., noise, air pollution, chronic stress) may influence disease onset or severity, yet robust study designs to disentangle these interactions are lacking.

To systematically explore this research gap, a three-part translational framework is designed. First, a prospective birth cohort would enroll children carrying known TS-associated risk genotypes (such as SLITRK1 or HDC variants) and follow them from the prenatal period through adolescence. Detailed prenatal and early-life exposure data (including fine particulate matter [PM2.5] levels and serial maternal cytokine profiles) would be collected and correlated with epigenetic modifications (for example, SLITRK1 promoter methylation) and neurodevelopmental outcomes. *In vivo* neuroimaging markers—such as striatal GABA concentration measured by magnetic resonance spectroscopy—would provide objective neural correlates. This design builds on evidence linking air pollution to neurotransmitter dysregulation relevant to TS, but will require careful sample stratification to ensure adequate numbers of variant carriers, long-term follow-up, and rigorous ethical oversight for pediatric genetic research. Second, geospatial analysis combined with machine learning could identify high-risk environments and gene–environment signatures. By integrating neighborhood-level pollution maps, individual gut microbiome profiles (via shotgun metagenomics), and psychosocial stress indicators (e.g., exposure to bullying), AI models may reveal patterns such as HDC variant carriers in heavily polluted areas developing early pharmacoresistance—a phenomenon suggested by studies in other inflammatory disorders. This approach demands large, multi-modal datasets and must address confounding factors and data privacy concerns. Finally, hypothesis-driven intervention trials would test causality and therapeutic strategies. For example, SLITRK1 variant carriers with gut dysbiosis could be enrolled in a randomized trial of the probiotic *Bifidobacterium longum* to assess effects on tic severity and microbial metabolites (such as butyrate). Similarly, HDC-variant subjects in high-pollution regions might receive combined interventions (HEPA air purifiers and histamine H3 receptor antagonists) to mitigate neuroinflammatory pathways implicated in TS. These trials would include rigorous controls, safety monitoring, and ethical safeguards appropriate for pediatric populations, with outcomes assessed at both clinical and molecular levels. Together, this integrative framework links genetic, environmental, and mechanistic perspectives on TS and provides a feasible roadmap for translational research from bench to bedside in neurology.

In conclusion, although genetic research has significantly advanced our understanding of TS, its practical impact depends on transcending disciplinary boundaries. Insights from ADHD research (82), where integration of genetic data with educational outcomes enabled personalized interventions, illustrate the potential of such interdisciplinary approaches. For TS, bridging genomic insights with real-world measures may clarify why the same genetic variant manifests as tics in some individuals and obsessive thoughts in others. Ultimately, this integration can transform molecular discoveries into meaningful improvements in patient care.

### 4.5 Treatment modalities in TS

A 2024 meta-analysis (83) demonstrated that repetitive transcranial magnetic stimulation (TMS) targeting the supplementary motor area or prefrontal cortex yields modest yet sustained tic reduction in TS, with mild, transient headache reported as the most common adverse effect; the authors recommend the adoption of standardized TMS protocols and extended follow-up to assess long-term efficacy. For refractory cases, a recent Cochrane review (84) confirmed that deep brain stimulation (DBS)—primarily directed at the centromedian–parafascicular thalamic complex—produces significant and durable symptom improvement, although patient selection criteria and optimal stereotactic targets warrant further refinement. Complementing these findings, a 2024 clinical series (85) reported that combining DBS with anterior capsulotomy provides additional benefit for patients with severe psychiatric comorbidities, albeit necessitating careful monitoring of postoperative cognitive and emotional outcomes. Meanwhile, a recent review of gut microbiome interventions in children (86) suggests that probiotic supplementation and diet-based strategies aimed at restoring microbial balance may help alleviate tic severity and comorbid behaviors, though larger randomized controlled trials are required to validate these preliminary observations and identify precise microbial targets. In the pharmacological domain, an updated review (87) reaffirms the use of *α*₂-adrenergic agonists (e.g., clonidine, guanfacine) as first-line agents and antipsychotics (e.g., aripiprazole, risperidone) as second-line options, while emerging therapies—including VMAT2 inhibitors and cannabinoid-based compounds—show promise in early-phase studies for reducing tics with potentially fewer metabolic side effects.

### 4.6 Advantages over prior bibliometric analyses and clinical implications

Compared with earlier, narrower-scope studies of TS literature (15), our work extends the temporal window from 1960 to 2024—capturing foundational milestones such as the introduction of DSM-III, the rise of genome-wide linkage studies, and pandemic-related publication shifts—rather than focusing solely on a single decade. Methodologically, we combine R/Biblioshiny, Tidyverse visualizations, VOSviewer network mapping, and Bradford’s Law to provide a multifaceted portrait of research trends, core journals, and author impact, whereas prior analyses relied primarily on CiteSpace. Rigorous data refinement—including iterative search optimization documented in a detailed flowchart and systematic imputation of missing references, affiliations, DOIs, and impact factors—ensures a reproducible dataset of 4,011 records. Beyond descriptive mapping, we introduce a tripartite translational framework that links genetic discoveries (SLITRK1, HDC) with environmental exposures and targeted intervention trials, offering a roadmap for precision prevention.

Clinically, these enhancements guide the formation of multinational TS consortia with more inclusive recruitment strategies, highlight validated assessment tools and novel therapies (e.g., VMAT2 inhibitors, digital biomarkers), and lay the groundwork for prospective G × E cohort studies. By integrating bibliometric insights with actionable trial designs, our study not only charts the evolution of TS research but also directly informs the development of personalized diagnostics and early-intervention strategies that can ultimately improve patient outcomes.

### 4.7 Limitations

This study has several limitations that may affect the comprehensiveness and representativeness of the findings. First, the analysis was restricted to the Web of Science (WoS) Core Collection, which, despite its broad coverage, may exclude relevant studies indexed in other databases, potentially overlooking regional or discipline-specific contributions. Second, the search was limited to publications from 1960 to 2024, which may have excluded earlier studies and the most recent preprints. Third, only English-language literature was included, introducing a potential linguistic and geographic bias by underrepresenting non-English research outputs. Additionally, the inclusion criteria were limited to articles and review articles, which may have excluded other meaningful publication types such as conference proceedings, editorials, or clinical guidelines. The use of a specific search strategy focused on Tourette syndrome may also have led to the exclusion of related research on comorbid conditions, such as ADHD or OCD, that did not explicitly mention TS in the title or abstract. These constraints may have influenced the observed patterns in journal distribution, author affiliations, and thematic evolution. Future research should expand the database scope (such as Scopus, PubMed, CNKI, and SciELO), incorporate multilingual and multi-format inclusion strategies, and refine search approaches to better capture the interdisciplinary and comorbid dimensions of TS research.

## 5 Conclusion

This analysis outlines the evolution of TS research from symptom-focused studies (1960s) to today’s integration of genetics, environmental triggers, and patient-centered care. The United States has led global efforts, with *JAMA Psychiatry* and pioneers like Yale’s Dr. Leckman shaping key insights. Future efforts must prioritize multidisciplinary collaboration integrating neuroimaging, environmental data, and AI, while advancing genetic insights to refine personalized therapies. Building inclusive global cohorts will ensure discoveries translate into equitable solutions, bridging research with clinical and societal needs.

## Data Availability

The datasets presented in this study can be found in online repositories. The names of the repository/repositories and accession number(s) can be found in the article/supplementary material.
